# 399. Recent COVID-19 infection is associated with high risk of mortality among patients with Staphylococcus aureus bacteremia

**DOI:** 10.1093/ofid/ofad500.469

**Published:** 2023-11-27

**Authors:** Emily Eichenberger, Allison Pall, Stepy Thomas, Susan M Ray

**Affiliations:** Emory School of Medicine, Atlanta, Georgia; Emory School of Medicine, Atlanta, Georgia; Emory University, Atlanta, Georgia; Emory University School of Medicine, Atlanta, Georgia

## Abstract

**Background:**

*Staphylococcus aureus* bacteremia (SAB) is associated with significant mortality. Recent reports indicate that the incidence of invasive *S. aureus* infections has increased since the start of the COVID-19 pandemic. The impact of recent COVID-19 infection on clinical outcomes of patients with SAB is unknown.

**Methods:**

We performed a retrospective study investigating outcomes associated with SAB in patients with recent COVID-19 infection (SAB-C) versus patients with SAB without a recent positive COVID-19 test (SAB-N). We used data collected by the Georgia Emerging Infections Program (EIP), a CDC- funded invasive *S. aureus* surveillance study in the 8-county Atlanta metro area to identify all patients with SAB between 2020-2021. SAB cases were linked to COVID-19 test results from the Georgia Notifiable Disease Reporting data to collect patient COVID-19 test results within 90-days of the initial positive blood culture. For subjects experiencing > 1 episode of SAB, only the first episode was evaluated to ensure independence of observations. Comparisons were made using Kruskal-Wallis test and Fisher’s exact test as appropriate. Statistical significance was set at P=0.05.

**Results:**

Between 2020 -2021 there were a total of 3705 unique patients with SAB. Among them, N=486 (13.1%) had a positive COVID-19 test <90 days prior to SAB (SAB-C), and N=3219 (86.9%) did not (SAB-N) (Table 1). Median time from positive COVID-19 test to first positive blood culture was 7 days. SAB-C had a greater proportion of methicillin resistant *S. aureus* (MRSA) and hospital onset infection than SAB-N (41.8% vs 36.1%, p=0.0189, and 45.7% vs 17.3%, p< 0.0001 respectively). 7-day mortality and in-hospital mortality were significantly higher among SAB-C vs SAB-N (25.7% vs 9.0%, p< 0.0001, and 43.6% vs 15.3%, p< 0.0001, respectively). Among SAB-C, hospital onset of SAB was significantly associated with mortality (29.6% survivors vs 66.5% non-survivors, p< 0.0001, Table 2).

**Figure:**
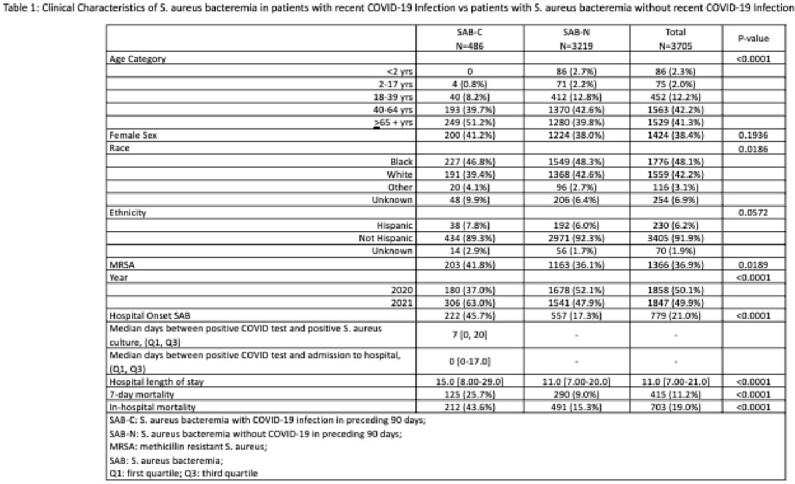

**Figure:**
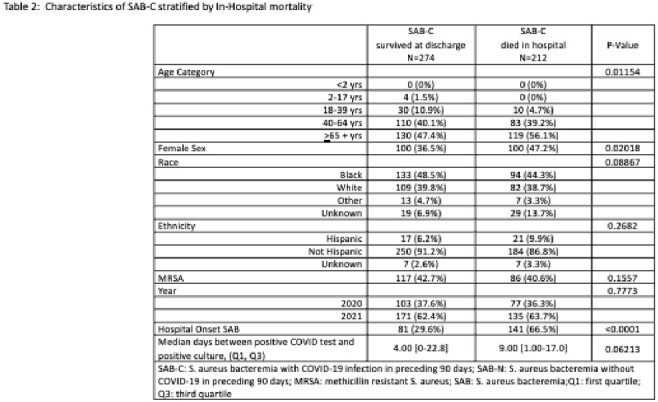

**Conclusion:**

SAB-C is associated with high mortality. Further research is needed to understand additional factors contributing to this high mortality rate including source of infection, infecting strain, and underlying host immune factors.

**Disclosures:**

**All Authors**: No reported disclosures

